# MISEV and MIQE: integrating domain-specific and general standards to strengthen extracellular vesicle biomarker research

**DOI:** 10.20517/evcna.2025.68

**Published:** 2025-10-24

**Authors:** Michael W. Pfaffl, Mikael Kubista, Jo Vandesompele, Stephen A. Bustin

**Affiliations:** ^1^Animal Physiology and Immunology, School of Life Sciences Weihenstephan, Technical University of Munich, Freising 85354, Germany.; ^2^Institute of Biotechnology CAS, Czech Academy of Science, Prague 252 50, Czech Republic.; ^3^Department of Biomolecular Medicine, Ghent University, Ghent 9000, Belgium.; ^4^Medical Technology Research Centre, Anglia Ruskin University, Chelmsford CM1 1SQ, UK.

**Keywords:** MIQE, MISEV, extracellular vesicles, biomarker research, reproducibility, standardization, analytical validity, RT-qPCR

## Abstract

Extracellular vesicles (EVs) have significant potential as therapeutic agents and as sources of diagnostic, predictive, and prognostic nucleic acid biomarkers. However, variability in EV workflows and inadequate standardization of downstream analysis pose major obstacles to reproducibility. The MISEV (Minimal Information for Studies of Extracellular Vesicles) guidelines provide essential domain-specific recommendations for EV isolation, characterization, analysis, nomenclature, and reporting, but deliberately refrain from prescribing methods for the molecular quantification of EV cargo. Among the analytical platforms used in EV studies, quantitative reverse transcription PCR (RT-qPCR) is the most critical method for validating and quantifying EV-associated RNA. The recently revised MIQE (Minimum Information for Publication of Quantitative Real-Time PCR Experiments) 2.0 guidelines offer a detailed foundation for ensuring analytical validity in RT-qPCR-based quantitative applications. The proposed model of harmonizing general and domain-specific guidelines provides a scalable blueprint for improving reproducibility across complex biomarker development workflows in molecular diagnostics.

## INTRODUCTION

Extracellular vesicles (EVs) are nanoscale, membrane-bound particles actively released by cells into the extracellular space. They encapsulate and protect molecular cargo such as proteins, lipids, and nucleic acids that reveal the physiological or pathological state of their cell of origin. Through their role in intercellular communication, EVs have emerged as a powerful source of circulating biomarkers^[1,2]^. Their diagnostic potential spans oncology, neurology, infectious diseases, fibrosis, and beyond^[2]^. However, the technical complexity of isolating and characterizing EVs from diverse biofluids compromises reproducibility across studies, raising concerns about the validity of reported biomarkers and the feasibility of clinical translation^[3,4]^.

To address these challenges, the International Society for Extracellular Vesicles (ISEV) developed the MISEV (Minimal Information for Studies of Extracellular Vesicles) guidelines, first published in 2014^[5]^, revised in 2018^[6]^, and most recently revised with a detailed methodological checklist^[7]^ and a comprehensive guide to EV research, biomarker discovery, and translational applications^[8]^. MISEV provides structured recommendations for EV characterization, including the use of molecular markers, biophysical analysis, high-resolution imaging, and functional assays. Although MISEV deliberately does not prescribe specific methodologies for nucleic acid quantification, its widespread citation and incorporation into community initiatives such as EV-TRACK suggest that these guidelines have promoted increased transparency and consistency in EV research and diagnostic applications^[9]^.

## EV CARGO QUANTIFICATION AND THE ROLE OF QUANTITATIVE REVERSE TRANSCRIPTION PCR

A variety of platforms are used to analyze EV-associated nucleic acids, including microarrays, reverse transcription PCR (RT-PCR), and RNA sequencing. Among these, reverse transcription quantitative PCR (RT-qPCR) is most widely used for validating candidate RNA biomarkers due to its sensitivity, specificity, and compatibility with clinical sample volumes. However, the reliability of RT-qPCR results depends critically on multiple factors such as primer design, RNA extraction, reverse transcription efficiency, data normalization, and statistical analysis. Minor variations in these steps can lead to significant discrepancies in reported outcomes^[10]^.

The MIQE (Minimum Information for Publication of Quantitative Real-Time PCR Experiments) guidelines offer a comprehensive framework for analytical rigor and reproducibility of qPCR workflows. Originally published in 2009^[11]^ and recently revised^[12]^, MIQE promotes methodological transparency and reproducibility in qPCR-based experiments. It covers critical aspects such as RNA quality assessment, RT conditions, primer specificity, amplification efficiency, and data normalization strategies. When rigorously applied, MIQE-compliant workflows reduce technical noise and support accurate, reproducible, and biologically meaningful data interpretation. Recent studies have also advanced the analytical rigor of RT-qPCR in EV research by introducing tools such as panels of stably expressed reference genes across diverse EV populations in liquid biopsy^[13,14]^ and recombinant EVs engineered to contain exogenous RNA, which serve as spike-in controls for assay validation and method comparison^[15]^. These innovations align with MIQE principles and further enable accurate and reproducible quantification of EV-associated RNA. While high-throughput techniques such as RNA sequencing have greatly expanded the discovery of novel potential biomarkers such as microRNAs, long non-coding RNAs, circular RNAs, and miscellaneous fragmented RNAs^[16]^, these methods require robust, independent validation. In most research studies, validation is performed using RT-qPCR, utilizing the methodological robustness encapsulated in MIQE. Thus, MIQE principles are central not only to primary data generation but also for validation of discoveries^[17]^.

## INTEGRATING MIQE INTO THE MISEV FRAMEWORK

The two guidelines are inherently complementary: MISEV addresses pre-analytical and EV-specific considerations, while MIQE defines best practices for nucleic acid quantification and transparent data reporting. The MISEV guidelines were developed under the auspices of the “ISEV” through dedicated working groups, community-wide consultation, and iterative revisions in 2018 and 2023^[5,6,7]^, while MIQE emerged from an international consortium of qPCR experts following focused workshops and collaborative discussions, with MIQE 2.0 reflecting technological and analytical advances since the original 2009 publication^[11,12]^. Earlier ISEV position papers provided complementary guidance, including recommendations on EV RNA analysis and bioinformatics^[18]^ and on the standardization of sample collection, isolation, and analysis^[19]^. These documents further reinforce the rationale for harmonizing MIQE and MISEV principles. Table 1 distinguishes parameters specific to MISEV, those unique to MIQE, and those addressed by both. This structure underscores that while MISEV provides domain-specific recommendations for EV isolation and characterization, it also includes guidance relevant to nucleic acid analysis, notably the quantification of total RNA (MISEV2023 §5.5) and nucleic acid characterization (MISEV2023 §6.5). MIQE, in contrast, defines the methodological details required for robust RT-qPCR assay design, validation, and transparent reporting. Areas of overlap include RNA quality control, assay validation, and data reporting, where adherence to both frameworks is essential for reproducibility.

**Table 1:** Scope and positioning of MISEV and MIQE guidelines in the EV analysis pipeline

**Stage in EV workflow**	**MISEV-specific**	**MIQE-specific**	**Both**	**Example parameters**
Pre-analytical	EV source description (biofluid, cell type); isolation method (ultracentrifugation, size exclusion chromatography, precipitation, *etc.*); removal of contaminants; storage conditions	Not directly addressed	Transparent reporting of sample provenance, handling, and storage	Volume and type of starting material; storage temperature and duration; processing time
Characterization	Particle size, distribution and concentration (NTA, TRPS, TEM); protein markers (EV positive and negative, tissue or cancer specific); imaging; biochemical composition	Not directly addressed	Documentation of EV purity and integrity alongside downstream molecular assays	Particle concentration units; vesicle morphology images; marker expression profiles
Molecular assay preparation	MISEV2023 §5.5: quantification of total RNA; MISEV2023 §6.5: nucleic acid characterization	RNA quality and quantity assessment; contamination checks	RNA input quantity, quality control, and contamination assessment required prior to downstream analysis	RNA integrity metrics (RIN/RQI); yield per vesicle/volume; DNA contamination checks
Assay design and validation	MISEV2023 §6.5: recognition of nucleic acid characterization, validation requirements	Primer/probe sequences; specificity checks; amplification efficiency; reverse transcription conditions	Transparency in assay design, validation of controls and standards, and overall reproducibility	Primer sequence disclosure; efficiency 90%-110%; linear dynamic range; RT protocol
Data acquisition	Not directly addressed	Reporting of Cq values; inclusion of controls (NTC, -RT); melt curves; technical/biological replicates	Use of appropriate controls, standards and full reporting of raw data	Raw Cq tables; replicate Cq SD; melt curve profiles
Data analysis and reporting	Transparent description of EV identity and purity	Normalization strategy; error metrics; statistical analysis; confidence intervals	Reporting of raw data tables, replicate statistics, and definition of technical *vs*. biological variability	Reference gene validation; normalization method; %CV on fold changes
Regulatory and clinical translation	Standardized EV terminology and provenance	Analytical validity documentation; reproducibility metrics; traceability	SOPs, inter-lab comparisons, reproducibility and transparency in clinical settings	SOPs for assay; inter-lab comparison results; reproducibility %CV

This table highlights the complementarity of MISEV and MIQE. The column entitled “MISEV-specific” captures EV-focused recommendations, the column entitled “MIQE-specific” details the RT-qPCR standards, and the column entitled “Both” identifies parameters jointly addressed. The last column shows example parameters. Together, these frameworks provide coverage from pre-analytical EV handling to downstream molecular quantification, ensuring methodological transparency and reproducibility. EV: Extracellular vesicle; MISEV: Minimal Information for Studies of Extracellular Vesicles; MIQE: Minimum Information for Publication of Quantitative Real-Time PCR Experiments; NTA: nanoparticle tracking analysis; TRPS: tunable resistive pulse sensing; TEM: transmission electron microscopy; RNA: ribonucleic acid; DNA: deoxyribonucleic acid; RIN: RNA integrity number; RQI: RNA quality indicator; RT: reverse transcription; Cq: quantification cycle; NTC: no template control; SD: standard deviation; CV: coefficient of variation; SOP: standard operating procedure.

Incorporating MIQE into the MISEV framework is therefore a logical extension, ensuring analytical rigor in the molecular quantification of EV-associated RNAs and maximizing reproducibility of the results. MISEV provides recommendations on how to assess the presence and purity of EVs using a combination of markers and biophysical approaches, but it does not teach how to reliably quantify the molecular cargo once the EVs are isolated. MIQE fills this gap by recommending a systematic evaluation of reverse transcription efficiency, assessment of PCR efficiency, and the use of validated reference genes for data normalization. These steps are essential to avoid technical biases that may obscure true biological differences^[20]^.

In addition, MIQE’s recommendations for structured assay design and transparent reporting support the reproducibility of EV transcriptomic data across platforms and laboratories. MIQE emphasizes the need to report primer sequences, reaction conditions, and raw quantification cycle (Cq) values, which aligns directly with MISEV’s emphasis on transparent reporting. Without this level of detail, even studies that adhere to MISEV’s isolation and characterization protocols may yield qPCR results that are not comparable among laboratories.

A third key area where MIQE supports MISEV goals is the validation of orthogonal methods. MISEV encourages the use of at least three different types of evidence, typically protein EV-specific surface markers, particle size measurements, high-resolution imaging, and the absence of protein or vesicular contaminants, to confirm EV identity. MIQE adds to this by recommending the inclusion of no-template controls, minus-RT controls, and melt curve analysis to ensure the specificity of the RT-qPCR assays used. These controls are particularly important in EV research, where low RNA input and contamination from non-vesicular sources can confound interpretation.

Finally, MIQE also addresses the often-overlooked issue of data analysis and interpretation. The guidelines recommend the use of appropriate statistical methods for both technical and biological replicates, and call for transparency in reporting error metrics, confidence intervals, and normalization strategies. This is particularly relevant in EV research, where biological variability from different liquid biopsies is often compounded by technical noise introduced during sample handling, storage, EV isolation, characterization, and quantification. Failure to account for these confounding factors can lead to misinterpretation of results, especially when distinguishing biologically meaningful signals from systematic bias and stochastic variation. By emphasizing statistical robustness and data quality, MIQE complements MISEV’s pre-analytical focus and helps ensure that derived conclusions are both reproducible and scientifically credible. The combined implementation of MIQE and MISEV thus extends beyond laboratory protocols, shaping a researcher’s laboratory mindset to value methodological consistency, rigorous analysis, and transparent reporting. This may be important for any kind of EV characterization or functional studies, but is essential for confidence in EV-based biomarker studies^[21]^.

Integrated MISEV-MIQE guidelines offer a comprehensive framework for EV-based molecular diagnostics: MISEV ensures the biological integrity and purity of vesicle preparations, and MIQE safeguards the analytical credibility of their molecular characterization. By formally recognizing this complementarity, the field can move toward a harmonized and reproducible standard of research that facilitates cross-study comparisons, meta-analyses, and clinical translation.

## TOWARD A GENERALIZABLE FRAMEWORK

While proposing here the complementary and logical integration of MIQE into a domain-specific guideline such as MISEV, this model can be extended to other application areas where (RT-)qPCR plays a central role - for example, microRNA research, circulating tumor DNA analysis, exRNA biomarkers, and single-cell and spatial transcriptomics. Each of these fields presents distinct challenges, but shares the need for standardized molecular workflows and would benefit from aligning field-specific practices with the principles of MIQE. While high-throughput approaches such as RNA sequencing are often used for biomarker discovery and exploratory research, downstream validation and quantification typically rely on RT-qPCR, where adherence to MIQE is essential to ensure analytical reliability and validity. More broadly, we argue that general standards such as MIQE should not be viewed as isolated technical checklists but as an adaptable framework that supports reproducibility across diverse biomedical contexts. Integrating such standards into domain-specific guidelines is a necessary step if molecular analysis is to address its reproducibility crisis and realize the full clinical potential of liquid biopsies and other emerging practices. Moreover, standardized reporting enables meaningful comparisons across studies, paving the way for robust meta-analyses, regulatory acceptance, and eventual integration into the clinical diagnostic workflows. This is particularly relevant in the context of multi-site collaborations, regulatory submissions, and diagnostic assay development, where the absence of methodological uniformity remains a significant barrier.

Beyond qPCR, the principle of integrating general and domain-specific standards is equally applicable to other platforms used in EV research, such as digital PCR (dPCR), immunoassays, and emerging microfluidic technologies. For dPCR, the digital MIQE (dMIQE) guidelines are available and offer a similar framework for integration^[22,23]^. A broader effort to contextualize these frameworks for EV research and other domains would promote consistency and cross-platform compatibility.

## LOOKING AHEAD - REGULATORY, EDUCATIONAL, AND TECHNOLOGICAL PERSPECTIVES

The integration of MIQE and MISEV is also highly relevant for regulatory bodies. As EV-based diagnostics advance toward clinical implementation, reproducibility, traceability, and transparency will be essential for regulatory approvals. Agencies such as the Food and Drug Administration (FDA) and European Medicines Agency (EMA) increasingly expect robust validation frameworks, including evidence of assay performance, standardized protocols, and transparent reporting. The combined MIQE-MISEV can facilitate this process by providing a harmonized foundation for assay development, method validation, and inter-laboratory comparisons. This is particularly important for clinical trials involving EV biomarkers, where consistent protocols are essential for achieving statistically and clinically meaningful results^[24]^.

A major challenge to the effective implementation of MIQE and MISEV lies in community-wide adoption. Although awareness of these guidelines is growing, many researchers remain unfamiliar with their full scope or perceive them as optional. Implementing both frameworks also introduces a greater burden in terms of experimental planning, documentation, and compliance. However, it is an investment essential to ensure the reproducibility and interpretability of EV biomarker data. Greater uptake will require coordinated efforts by stakeholders across academia, publishers, funding bodies, and educational bodies. Journals and funding bodies can support this process by encouraging or requiring guideline-compliant reporting, while integration into reviewer checklists and postgraduate curricula can embed good scientific practice from the outset. Transparent, standardized workflows not only improve data quality, but also enhance the credibility and translational value of EV-based molecular diagnostics.

As molecular diagnostics continues to evolve, the principles embodied by MIQE and MISEV must remain adaptable. Emerging technologies, including single-EV analysis, microfluidics, and multi-omic profiling, will introduce new sources of variability and analytical complexity. Rather than being replaced, existing guidelines must evolve to interface with these technologies, offering modular but coherent recommendations for new contexts. This flexibility will be essential as the field moves from bulk measurements to highly granular and integrated diagnostic approaches. The MIQE-MISEV model may therefore serve as a living template for building future-ready standards that can support both innovation and reproducibility.

## CONCLUSION

The growing interest in EVs as an important source of circulating nucleic acid biomarkers demands a parallel commitment to methodological standardization. MISEV has significantly advanced the field by establishing best practices for EV isolation and characterization. However, full reproducibility and analytical validity can only be achieved when downstream molecular quantification is held to equally rigorous standards. MIQE provides this necessary complement, offering a robust framework to ensure that qPCR-based analyses of EV-associated nucleic acids are scientifically sound, transparent, and reproducible. We propose that MIQE and MISEV be jointly adopted in EV biomarker discovery studies, with MIQE guiding analytical processes and MISEV anchoring pre-analytical integrity. Together, they reinforce the entire experimental pipeline, enabling more reliable cross-study comparisons, supporting regulatory validation, and accelerating clinical translation. As the field moves toward increasingly complex and data-rich clinical applications in liquid biopsy, such an integrated approach is both timely and essential. Standardized, transparent, and reproducible workflows are the foundation of credible biomarker discovery and are critical for translating laboratory insights into clinical realities. This model of harmonizing general and domain-specific guidelines provides a scalable blueprint for improving reproducibility across complex biomarker development workflows in molecular diagnostics.

## DECLARATIONS

### Copyright

© The Author(s) 2025.
